# Correction: Genetic differentiation at extreme latitudes in the socially plastic sweat bee *Halictus rubicundus*

**DOI:** 10.1371/journal.pone.0317155

**Published:** 2025-01-03

**Authors:** Bas A. Michels, Mariska M. Beekman, Jeremy Field, Jodie Gruber, Bart A. Pannebakker, Charlotte Savill, Rebecca A. Boulton

The Fig 2 is uploaded incorrectly. Please see the correct Fig 2 here.

**Fig 2:**
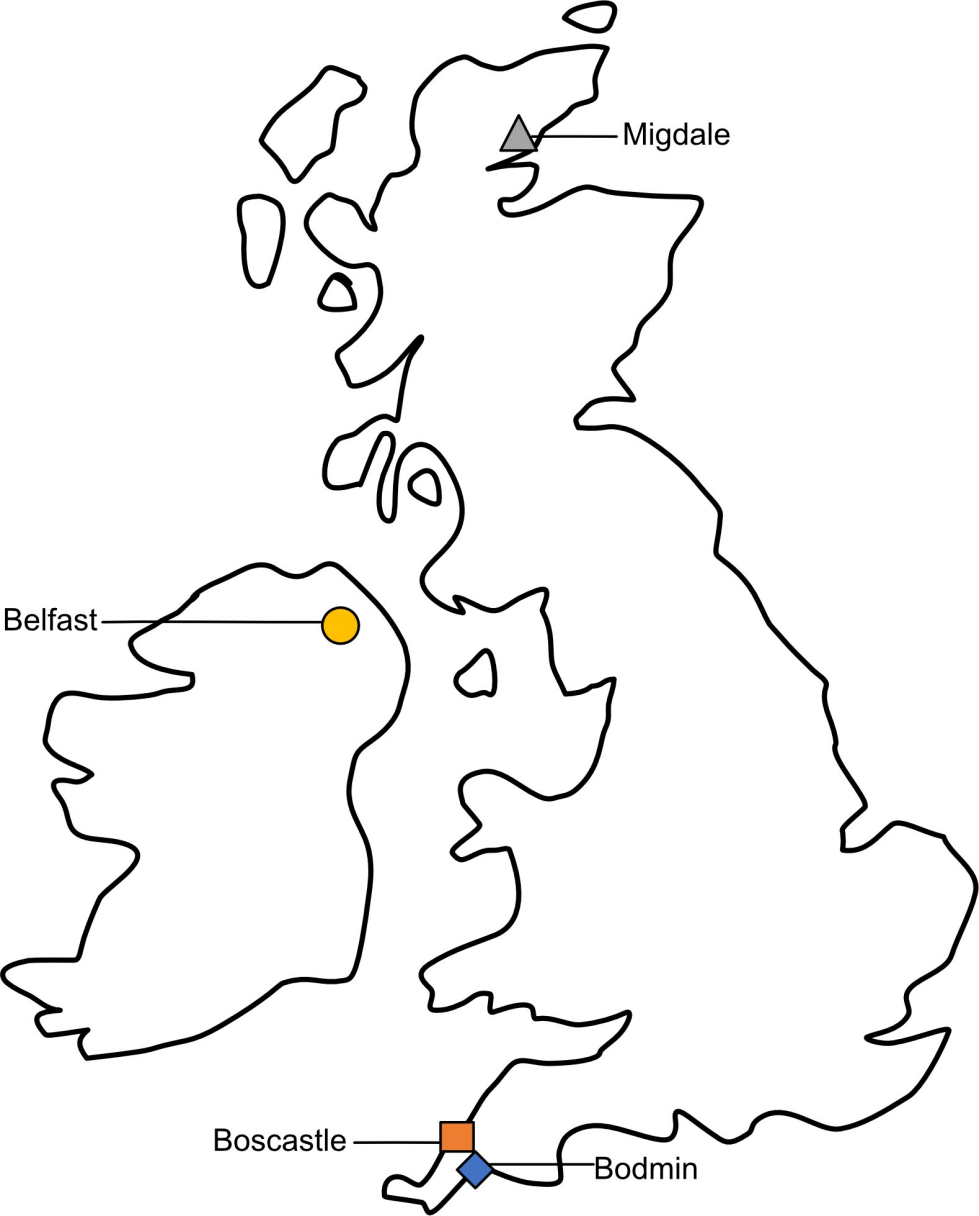
The sampling locations of the *Halictus rubicundus* aggregations used in this study.

This image was reproduced from [7] under the terms of the Creative Commons Attribution licence (CC BY).
