# Supplementary material for: Genetic differentiation at extreme latitudes in the socially plastic sweat bee Halictus rubicundus
Source: PLoS One. 2024 May 29;19(5):e0302688. doi: 10.1371/journal.pone.0302688 (PMC11135698; doi:10.1371/journal.pone.0302688)
Supplement: S1 Table — (DOCX) [file pone.0302688.s001.docx]

| Set | Primer name | Range | Dye colour | GenBank  accession number | Primer sequences (5’-3’) (F primers are labelled on 5’ end) |
| --- | --- | --- | --- | --- | --- |
| S1 | rub02 | 168-190 | 6-FAM (blue) | BV729095 | F: CCAGCCGGCCAACGTTGC R: CGGAGCTGAAAACTCAATTACAG |
| S1 | rub30 | 150-200 | NED (yellow) | BV729098 | F: GATCCGCTTTCAACCGTCCG  R: GTGAGCTGGGTCCGGCGAG |
| S1 | rub35 | 177-273 | PET (red) | BV729108 | F: GATGACGCAGTACGAACGG  R: GCTTCGACGTATGATTATCC |
| S1 | rub37b | 88-94 | 6-FAM (blue) | BV729099 | F: GATTTTTCTCGCGTACCTCTGC  R: CACTGCGATTCCGGGTTGTCC |
| S1 | rub59 | 185-211 | VIC (green) | BV729101 | F: GTGACCAGGTGCGCTCGTTAC R: CCGTGTCCCCAGCTCCGTTTC |
| S2 | rub04 | 197-205 | PET (red) | BV729096 | F: CGGACGTTTTTCAATGTTTTTC  R: CGTCCGACTGCATTCTCTTTG |
| S2 | rub06 | 238-284 | 6-FAM (blue) | BV729097 | F: GTCTGGCGGAAGTCTACGTGC  R: CAAGTTCGGTGCGTTAGATAATG |
| S2 | rub55 | 133-155 | NED (yellow) | BV729100 | F: GCTATAAAAGGCGAAACGGGTG R: CTCCTATCCGGTTGACATTGCC |
| S2 | rub60 | 123-149 | 6-FAM (blue) | BV729102 | F: GCAAACACACCGCTAATGACATG R: GCCGACAGGTTTGCAGCATGAG |
| S3 | rub73 | 165-195 | NED (yellow) | BV729105 | F: GCTTTGTTTCTCACTATCGTCCC  R: CGCGCAAAGTTCCCAGGGGTG |
| S3 | rub80 | 100-180 | VIC (green) | BV729107 | F: CCGGTCGGAGGTGTGTTC R: CATACTTCCTTCCTAGCATTCG |
| S3 | rub61 | 180-202 | PET (red) | BV729103 | F: GACGCGGAAATAGAAAAGTTG R: CTAATGCATCGGGCCAACTG |
| S3 | rub72 | 149-177 | 6-FAM (blue) | BV729104 | F: GCATTTATTCCGTCGCGACTC R: CGGTGGCGGGGCTCGTAATG |

**Table S1: Details of the primers used in each of the 3 sets.**
