# Supplementary material for: Genetic differentiation at extreme latitudes in the socially plastic sweat bee Halictus rubicundus
Source: PLoS One. 2024 May 29;19(5):e0302688. doi: 10.1371/journal.pone.0302688 (PMC11135698; doi:10.1371/journal.pone.0302688)
Supplement: S2 File — (DOCX) [file pone.0302688.s003.docx]

## Supplementary information

**Table S1: Details of the primers used in each of the 3 sets**

used.

| Set | Primer name | Range | Dye colour | GenBank  accession number | Primer sequences (5’-3’) (F primers are labelled on 5’ end) |
| --- | --- | --- | --- | --- | --- |
| S1 | rub02 | 168-190 | 6-FAM (blue) | BV729095 | F: CCAGCCGGCCAACGTTGC R: CGGAGCTGAAAACTCAATTACAG |
| S1 | rub30 | 150-200 | NED (yellow) | BV729098 | F: GATCCGCTTTCAACCGTCCG  R: GTGAGCTGGGTCCGGCGAG |
| S1 | rub35 | 177-273 | PET (red) | BV729108 | F: GATGACGCAGTACGAACGG  R: GCTTCGACGTATGATTATCC |
| S1 | rub37b | 88-94 | 6-FAM (blue) | BV729099 | F: GATTTTTCTCGCGTACCTCTGC  R: CACTGCGATTCCGGGTTGTCC |
| S1 | rub59 | 185-211 | VIC (green) | BV729101 | F: GTGACCAGGTGCGCTCGTTAC R: CCGTGTCCCCAGCTCCGTTTC |
| S2 | rub04 | 197-205 | PET (red) | BV729096 | F: CGGACGTTTTTCAATGTTTTTC  R: CGTCCGACTGCATTCTCTTTG |
| S2 | rub06 | 238-284 | 6-FAM (blue) | BV729097 | F: GTCTGGCGGAAGTCTACGTGC  R: CAAGTTCGGTGCGTTAGATAATG |
| S2 | rub55 | 133-155 | NED (yellow) | BV729100 | F: GCTATAAAAGGCGAAACGGGTG R: CTCCTATCCGGTTGACATTGCC |
| S2 | rub60 | 123-149 | 6-FAM (blue) | BV729102 | F: GCAAACACACCGCTAATGACATG R: GCCGACAGGTTTGCAGCATGAG |
| S3 | rub73 | 165-195 | NED (yellow) | BV729105 | F: GCTTTGTTTCTCACTATCGTCCC  R: CGCGCAAAGTTCCCAGGGGTG |
| S3 | rub80 | 100-180 | VIC (green) | BV729107 | F: CCGGTCGGAGGTGTGTTC R: CATACTTCCTTCCTAGCATTCG |
| S3 | rub61 | 180-202 | PET (red) | BV729103 | F: GACGCGGAAATAGAAAAGTTG R: CTAATGCATCGGGCCAACTG |
| S3 | rub72 | 149-177 | 6-FAM (blue) | BV729104 | F: GCATTTATTCCGTCGCGACTC R: CGGTGGCGGGGCTCGTAATG |

S2 Extended methods

**Peak scoring**
In this research project the values of each peak within this Geneious file were scored in Geneious v. R11 (<https://www.geneious.com>) in a marker-focused manner (i.e. looking at a single microsatellite marker for all individuals before moving on to the next marker). Scored values represent the number of microsatellite repeats for each marker, different values for the same microsatellite marker represent different alleles. Peaks were scored for quality and some individuals were scored multiple times to ensure consistency. Some individuals were genotyped a second time if initial PCRs partially or fully failed to amplify DNA or if reliable scores could not be determined due to stutter around peaks or other common issues with repeatability

While scoring the peaks it was important to determine which of the peaks represents the allele and which are noise. A peak needs to meet several requirements to be scored as the allele peak. It should be distinct from the background. In the case of heterozygosity, the second peak should be lower than the first peak. Smaller peaks lead up to the final peak. These leading peaks are a good inclination that the final peak of that series represents the allele (figure S1). Small peaks in front of the allele peak can be explained due to adenylation.

**Figure S1:** What leading peaks look like in Geneious [22].

As these microsatellites were created over a decade before this experiment was performed, it is to be expected that some of the peak ranges have shifted. This was true with almost all locus ranges, which were thus altered if a lot of obvious peaks were found out of range in multiple individuals. The S1 blue locus range contained two markers in the same colour. Alteration of these locus ranges were handled with extra caution, as I could have accidentally increased the range in such a way that an allele from the first marker would have been scored in the second one.
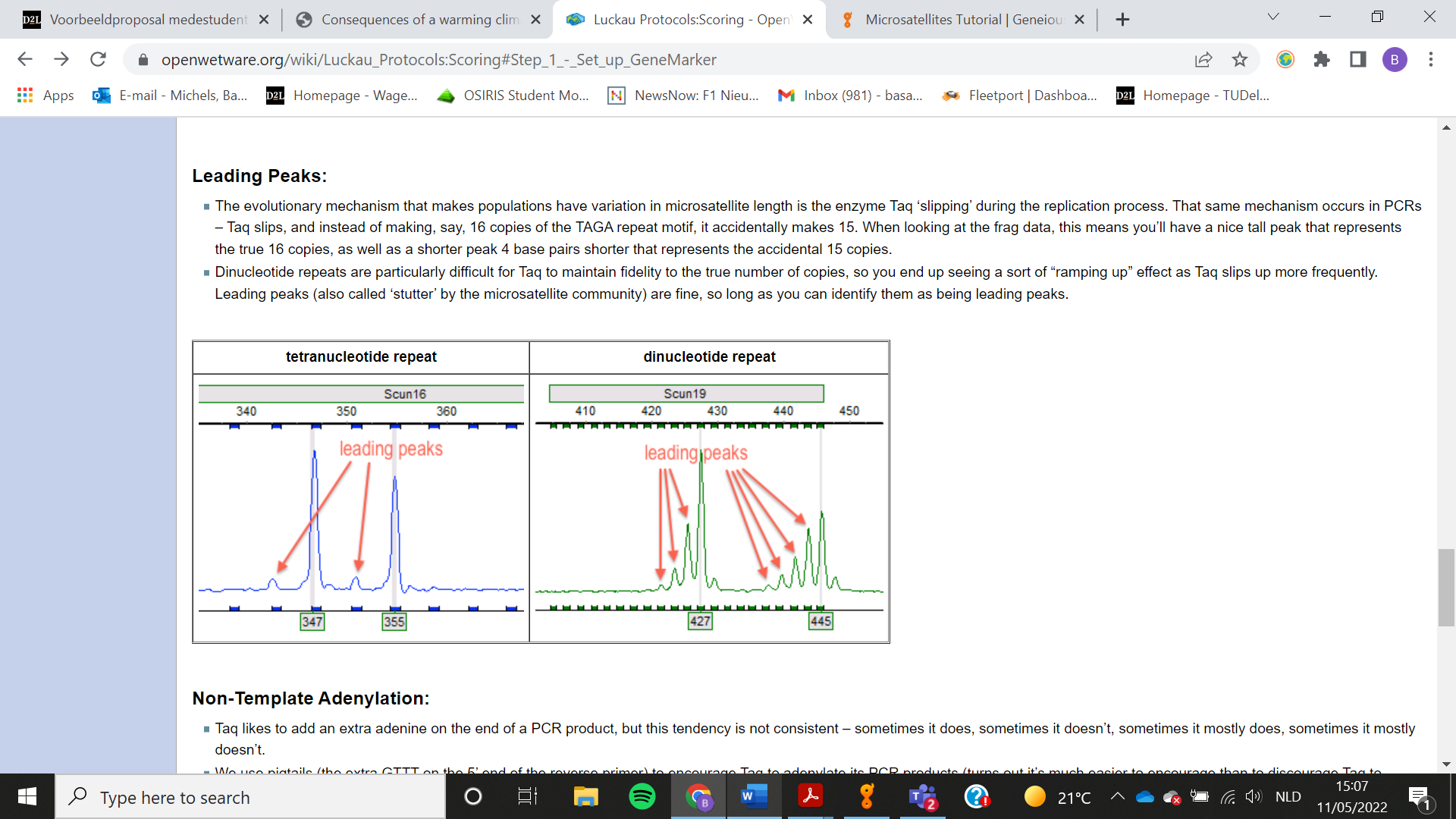
.

**GeneAlEx analyses**
Nei’s unbiased genetic distance may give spurious results when homozygosity and sample size is small, so it was decided to use the normal Nei’s genetic distance.

Several analyses were performed via GeneAlEx, which were calculated in the following way.
F_ST_ is the a measure of population differentiation due to genetic structure. F_ST_ = (H_E_ – H_O_)/H_E_.
H_O_ is the observed heterozygosity. H_O_ = Number of heterozygotes / Total number of individuals
H_E_ is the expected heterozygosity. H_E_ = 1 – Sum pi^2^. Where pi is the frequency of the i^th^ allele for the population and Sum pi^2^ is the sum of the squared population allele frequencies.
Nei’s genetic distance is another measure similar to Fst. Both are true in our case, so this wasn’t used.

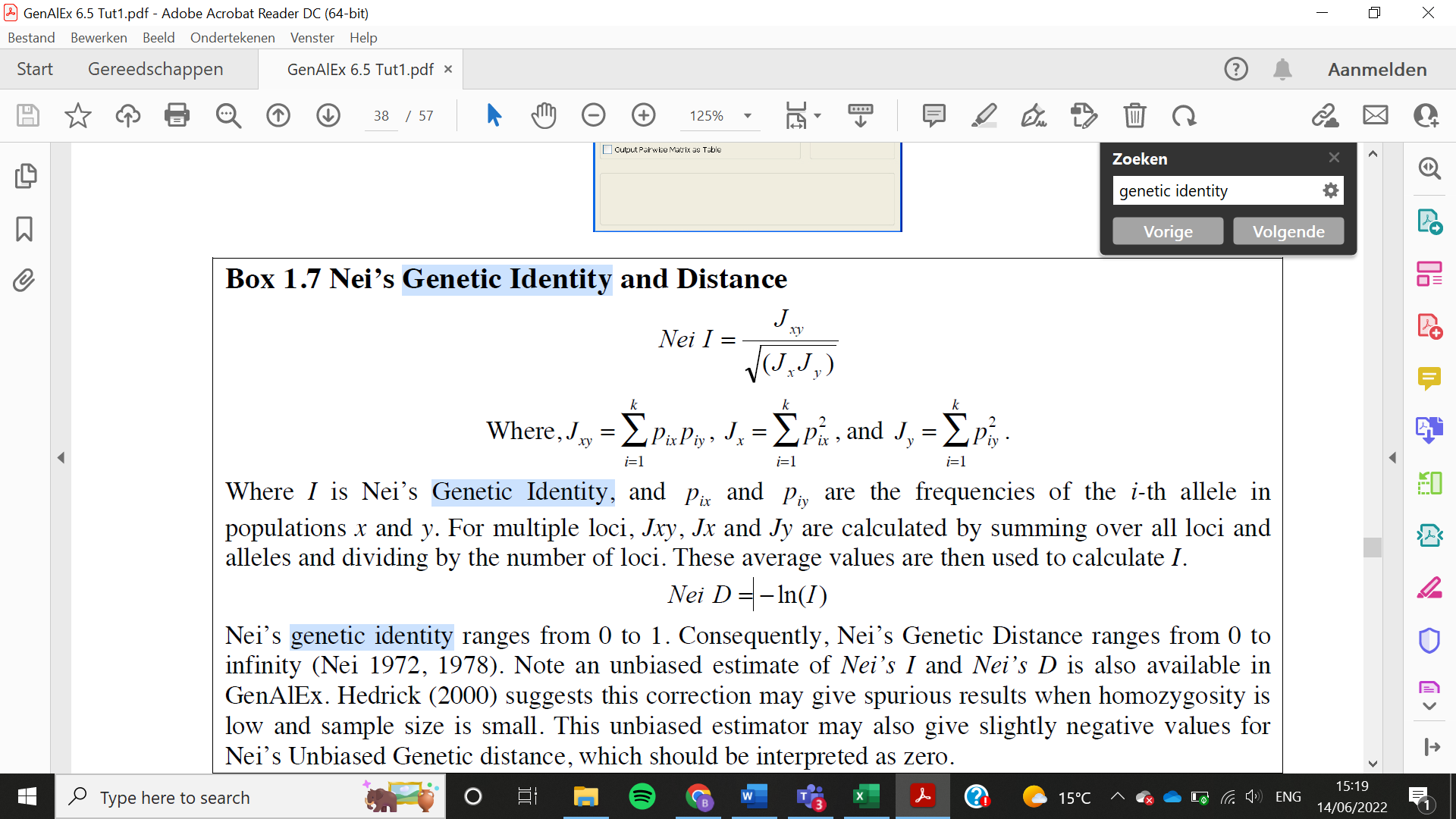

Where *I* is Nei’s Genetic Identity, and *p_ix_* and *p_iy_* are the frequencies of the *i-*th allele in populations *x* and *y.* For multiple loci, *J_xy_*, *J_x_* and *J­_y_* are calculated by summing over all loci and alleles and dividing by the number of loci. These average values are then used to calculate *I*.
*Nei D* = -ln(*I*). Gives the Nei genetic distance value.
