# Supplementary material for: Genetic differentiation at extreme latitudes in the socially plastic sweat bee Halictus rubicundus
Source: PLoS One. 2024 May 29;19(5):e0302688. doi: 10.1371/journal.pone.0302688 (PMC11135698; doi:10.1371/journal.pone.0302688)
Supplement: Attachment — Submitted filename: PONE-D-23-27285_review.pdf [file pone.0302688.s004.pdf]

The paper presents the results of a study aiming to understand whether differences in phenology due to environmental conditions could limit gene flow in *Halictus rubicundus* bees. My impression is that the data are interesting and, with a few adjustments, would make a nice paper in PLOS ONE. Follow my comments:

1) In general, in the introduction, I think it misses a description of the types of barriers, in addition to the distance between populations, that could influence bee gene flow. The authors cite the Irish Sea and temperature differences, but between the south and north of the UK, there are possibly other barriers that could be better described. My knowledge about UK geography is poor, just like many readers abroad. So, the detail of this point is important to strengthen the work.

2) Lines 43–44: "*Halictus rubicundus* (Hymenoptera: Halictidae) is a locally abundant species with a broad geographic range". Where are the bees locally abundant? In the north of the globe? In UK? I understand your point, but starting the text with this information without giving a basis to understand it doesn't seem right.

3) Lines 205-207: "Migdale was the most genetically different from all other populations (Bodmin: FST = 0.061; Nei's = 0.618. Boscastle: FST = 0.059; Nei's = 0.625. Belfast: FST = 0.054; Nei's = 0.491)". I suggest adding the values of Migdale at this point in the text to facilitate the comparison.

4) Lines 207-210: "Bees collected in the south of mainland Britain (Bodmin and Boscastle) were more genetically similar to bees across the Irish Sea (Belfast) than they were to bees collected in the North of mainland Britain in Migdale (Bodmin: FST = 0.046; Nei's = 0.504. Boscastle: F ST = 0.044; Nei's = 0.503)." Again, please add the values of all points cited in the text.
