# Supplementary material for: Genetic differentiation at extreme latitudes in the socially plastic sweat bee Halictus rubicundus
Source: PLoS One. 2024 May 29;19(5):e0302688. doi: 10.1371/journal.pone.0302688 (PMC11135698; doi:10.1371/journal.pone.0302688)
Supplement: Attachment — Submitted filename: Response to Reviewers 17-12.docx [file pone.0302688.s005.docx]

Reviewer #1: The paper presents the results of a study aiming to understand whether differences in phenology due to environmental conditions could limit gene flow in Halictus rubicundus bees. My impression is that the data are interesting and, with a few adjustments, would make a nice paper in PLOS ONE. Follow my comments:

We thank reviewer #1 for these kind words.

1) In general, in the introduction, I think it misses a description of the types of barriers, in addition to the distance between populations, that could influence bee gene flow. The authors cite the Irish Sea and temperature differences, but between the south and north of the UK, there are possibly other barriers that could be better described. My knowledge about UK geography is poor, just like many readers abroad. So, the detail of this point is important to strengthen the work.

We agree that this part of the introduction can be extended, so we have also introduced the Scottish Highlands as a possible barrier between the south and north, and some other barriers (L97-103).  

2) Lines 43–44: “Halictus rubicundus (Hymenoptera: Halictidae) is a locally abundant species with a broad geographic range”. Where are the bees locally abundant? In the north of the globe? In UK? I understand your point, but starting the text with this information without giving a basis to understand it doesn’t seem right.

We have changed this sentence to be more specific (L38-40).

3) Lines 205-207: “Migdale was the most genetically different from all other populations (Bodmin: FST = 0.061; Nei’s = 0.618. Boscastle: FST = 0.059; Nei’s = 0.625. Belfast: FST = 0.054; Nei’s = 0.491)”. I suggest adding the values of Migdale at this point in the text to facilitate the comparison.

These values shown here are values comparing Migdale with Bodmin, Boscastle and Belfast. We agree see that this might not be clear from the way it was expressed, and have added a table (Table 4) displaying these values so there may be no misunderstanding of how to interpret the results.

4) Lines 207-210: “Bees collected in the south of mainland Britain (Bodmin and Boscastle) were more genetically similar to bees across the Irish Sea (Belfast) than they were to bees collected in the North of mainland Britain in Migdale (Bodmin: FST = 0.046; Nei’s = 0.504. Boscastle: F ST = 0.044; Nei’s = 0.503).” Again, please add the values of all points cited in the text.

This is the same issue as with comment #3, and we think the addition of the Table 4 will also be beneficial in the presentation of the data for these comparisons.

Reviewer #2: Article number PONE-D-23-27285 “Genetic differentiation at extreme latitudes in the socially plastic sweat bee Halictus rubicundus” carried results that need revision for the improvement of the draft. Moreover, the conclusion portion should be further developed based on the findings and future implications of the study. Some suggested changes are in the comments portion to revise and improve the manuscript. Please find suggested corrections, Reference writing, journal-style format, author’s instructions, use of abbreviations and missing information for revision.

We thank the reviewer for their feedback on our study and have adjusted the manuscript, based on their suggestions, as described below.

Abstract: Abstract is lengthy, please be precise in the abstract writings.

We have reduced the abstract significantly for conciseness.

Line 45: Please recheck the abbreviations H. rubicundus” and write complete scientific name at the start of the sentence.

We have written out the complete scientific name at the start of the sentence (L41).

Line 45-48: Please rephrase the sentence

We are unsure what the reviewer would like us to change about this sentence. We are happy to edit with further comments from the reviewer.  

Line 48: (the number of generations per year)” remove the sentence

This sentence has been removed (L43).

Line 53-54: (a single generation per 54 year)” please remove the sentence.

This sentence has been removed (L47).

Line 54: see also Hogendoorn and Leys 1997” please follow the author’s instructions and journal style format while writing the references” this may be recheck throughout the manuscript

https://www.researchgate.net/post/How_to_auto_convert_references_from_word_file_to_Mendeley

We thank the reviewer for pointing this out. We have changed the reference format accordingly.

Figure 1 may be placed as supplementary figure 1 (figure taken from Schürch et al. 2016) while mentioning the Figure S1 in the text.

We understand the point reviewer #2 is making here. However, we feel that Figure 1 is essential to the introduction, as a big part of this paper is about the life cycle of *H. rubicundus* and how differences could influence phenology and gene flow. Therefore, we believe that a Figure 1 is an important visual aid to set up the study and so we have left it as a main figure. We are happy to be guided by the editors preference with regards to moving Figure 1 to the supplementary material.

Material and methods 
The authors collected samples in 2019-2020 and conducted analysis? There is no data found regarding the background of the samples and even the generational data?

We thank the reviewer for raising this point. We have added this information to the data repository, for which the link can be found in the supplementary section. This is the link to the meta-data with details about collection of the samples <https://osf.io/pvj56?view_only=aebe7992b4cb4285a90a0e6eb2603685>

Line 160: HWE?

We have added ‘Hardy-Weinberg equilibrium’ in full (L159).

Line 249: to the” remove from the sentence

Done.

Please add a precise conclusion

We thank reviewer #2 for their feedback regarding our conclusion. We have added a more precise conclusion (L388-393).

Please write the references in the text and in the reference portion according to the author's instructions and Journal style/formatting. Please double-check for typos and inconsistencies in Journal style/formatting/ authors instructions, double spaces, spellings of the words, English vocabulary, missing italics, scientific names, missing information

Done.

Reviewer #3: This article presents a study on the genetic differentiation seen in different populations of Halictus rubicundus in the UK. The paper addresses an interesting topic and is well written; I don’t have comments about the analyses or the interpretation of the results. I feel the discussion could be fleshed out a bit, however, and so I recommend it for publication in PLOS One pending the minor revisions to the discussion outlined below.

We thank reviewer #3 for their kind words, and have fleshed out the discussion, specifically on future research recommendations, as per their suggestion.  
 
The results of the study uncover genetic differentiation between populations in the north and the south of the UK and the authors state that their results “improve understanding of barriers to gene flow”. While the authors suggest that this differentiation may be caused by both phenological and /or physical barriers to gene flow, the study does not appear to have made any attempt to separate these factors. To truly understand the barriers to gene flow, and thus the potential consequences of climate change or habitat degradation on these populations, separating phenological barriers from physical barriers seems essential. Perhaps it was not possible in the present study…but how could this be done in a future study? To highlight geographical barriers - would it be useful to increase the sampled nests to include populations from along the north-south gradient of the UK? If the Scottish Highlands truly represent a barrier to gene flow, then populations in the Scottish lowlands should also be quite different from the Migdale populations. The Irish Sea was not found to have been a total barrier to gene flow (Belfast populations overlapped quite a bit with the Cornish and, to a lesser degree, with the Migdale). Maybe this should be discussed in more detail, given what we know about dispersal capacities of species of bees across bodies of water? Or maybe there’s another explanation for this overlap, i.e. similar alleles that have been retained in different populations despite reproductive isolation? And to look at phenological barriers to gene flow - could one take into consideration voltinism when comparing genetic differentiation (i.e. is there more gene flow between populations that are either univoltine or bivoltine, and less among populations with different social structure?)
We agree with the suggestion of reviewer #3 to include populations from the Scottish lowlands (the central belt) in future comparisons, to test whether the Scottish highlands are indeed the main barrier to gene flow (L361-363).
Regarding the comment on the Irish Sea from reviewer #3, we still mention that the Irish Sea is a barrier to gene flow, just not as much as expected (L249-252). We think that it is very unlikely that the similarity is due to similar alleles being retained over time, as we are working with microsatellites here, which should be selectively neutral.
 
Finally, to the question "Have the authors made all data underlying the findings in their manuscript fully available?", I answered no because I couldn't find any mention of where the microsat markers generated in the study were stored...

We have added the raw unscored data files (.fsa format) which can now be accessed via the repository here <https://osf.io/s8txc/?view_only=aebe7992b4cb4285a90a0e6eb2603685>, as also described in the supporting information in the manuscript (L386-387).
