# Supplementary material for: Genetic differentiation at extreme latitudes in the socially plastic sweat bee Halictus rubicundus
Source: PLoS One. 2024 May 29;19(5):e0302688. doi: 10.1371/journal.pone.0302688 (PMC11135698; doi:10.1371/journal.pone.0302688)
Supplement: Attachment — Submitted filename: Response to Reviewers 18-3.docx [file pone.0302688.s006.docx]

Reviewer #2: The revised article, PONE-D-23-27285R1, "Genetic differentiation at extreme latitudes in the socially plastic sweat bee Halictus rubicundus" has improved after revision; however, some issues still need to be verified before the draft is officially considered.
The authors feel that “figure 1 is essential to the introduction, as a big part of this paper is about the life cycle of H. rubicundus and how differences could influence phenology and gene flow. Therefore, we believe that figure 1 is an important visual aid to set up the study and so we have left it as a main figure.......” Kindly recheck and consult for the journal's policies with the managing editor. "It is okay with me if the journal policies permit the reproduction of figure 1 from the earlier literature.

We have made our own version of the figure from Shurch et al, and this is now used as figure 1. We still credit the original paper in the legend due to the similar format.
The authors should also recheck the formatting guidelines for tables. Journal-style formatting and adjustments should be made as needed.

Done

Line 380: Reference [2] please recheck if this reference is relevant to the text (if necessary)

We have kept this reference in (now line 358 in the track changed version of the ms) this statement refers to the greater declines in pollinators in the north and this reference (where we originally read this) is the most appropriate.

Could you please confirm that the references in the discussion section follow the numerical sequence, as they appear to be out of order? (Make changes if necessary.)

Done

Please write the references in the text and in the reference portion according to the author's instructions and Journal's style and formatting. Please double-check for typos and inconsistencies in Journal style formatting, author's instructions, double spaces, spellings of the words, English vocabulary, missing italics, scientific names, and missing information etc.

Done
